# Serological Screening Suggests Presence of Schmallenberg Virus in Cattle, Sheep and Goat in the Zambezia Province, Mozambique

**DOI:** 10.1111/tbed.12234

**Published:** 2014-05-15

**Authors:** A-L Blomström, H Stenberg, I Scharin, J Figueiredo, O Nhambirre, A P Abilio, J Fafetine, M Berg

**Affiliations:** 1 Department of Biomedical Sciences and Veterinary Public Health, Section of Virology, Swedish University of Agricultural SciencesUppsala, Sweden; 2 Biotechnology Center, Eduardo Mondlane UniversityMaputo, Mozambique; 3 National Health InstituteMaputo, Mozambique

**Keywords:** emerging diseases, arboviruses, diagnostics, veterinary epidemiology, virus

## Abstract

Schmallenberg virus (SBV) is a novel *Orthobunyavirus* within the family *Bunyaviridae* belonging to the Simbu serogroup. Schmallenberg virus infects ruminants and has since its discovery in the autumn 2011 been detected/spread to large parts of Europe. Most bunyaviruses are arboviruses, and SBV has been detected in biting midges in different European countries, suggesting that they may play a role in the transmission of the virus. It is not known how SBV was introduced to Europe and if SBV is present in countries outside of Europe. Thus, in this study, we conducted a serological screening for SBV antibodies in cattle (no. 79), sheep (no. 145) and goat (no. 141) in the Zambezia Province in Mozambique during September 2013. The results show a high percentage of antibody-positive animals. All farms tested had seropositive animals; cattle displayed the highest prevalence with 100% positive animals. Sheep and goat also displayed high number of positive animals with a 43–97% and 72–100% within-herd seroprevalence, respectively. This initial serological screening suggests that SBV is present on the African continent. However, cross-reactivity with other members of the Simbu serogroup cannot be ruled out, and further studies are needed to identify and characterize the virus responsible for the antibody-positive results.

## Introduction

At the end of the summer of 2011, a non-specific syndrome in cattle including fever, diarrhoea and decreased milk production was reported in several farms in Germany and the Netherlands. A metagenomic analysis of blood samples from infected cattle identified a novel *Orthobunyavirus* belonging to the Simbu serogroup, and the virus was provisionally named Schmallenberg virus (SBV) after the town in Germany where the virus was first identified (Hoffmann et al., 2012). After the initial discovery, the virus has been detected in/spread to at least 20 countries in Europe including both southern European countries such as Italy and France as well as northern countries such as Sweden, Norway and Finland (EFSA, 2012). This was the first detection of a member of the Simbu serogroup in Europe as members belonging to this group have previously been detected only in Africa, Asia, the Americas and Australia. Schmallenberg virus has been shown to not only infect cattle but other ruminants, such as sheep, goat and deer, which were shown to be SBV positive (EFSA, 2012). Adult animals present, if any, often mild symptoms. However, transplacental infection can occur leading to abortion, stillborns and malformations of the lambs, calves and goat kids (Beer et al., 2013; Conraths et al., 2013). Almost all members of the *Bunyaviridae* family are arboviruses, that is, transmitted by an arthropod vector, such as mosquitoes, ticks and biting midges. The detection of SBV in biting midges (*Culicoides* spp.) in Denmark (Rasmussen et al., 2012, 2014), the Netherlands (Elbers et al., 2013a,b) and Italy (Goffredo et al., 2013) suggests that biting midges may play an important role in the transmission of the virus.

The origin of SBV is at present unknown as is the global distribution of the virus in ruminants in countries outside of Europe. Thus, in this study, we conducted a limited serological screening of SBV antibodies in cattle, sheep and goat in Mozambique in Africa.

## Material and Methods

**Figure 1:**
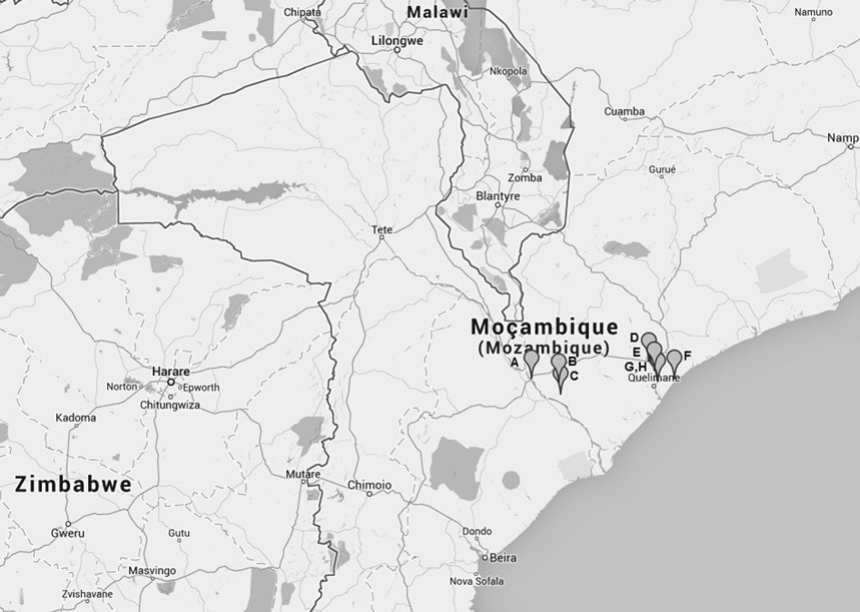
A map showing the position of the eight farms sampled in Zambezi Province, Mozambique. A – Mopeia Chimuara; B – Mopeia South; C – Mopeia Deda; D – Nicoadala Amed; E – Nicoadala Mucelo; F – Nicoadala Mingano; G – Quelimane Padeiro; and H – Quelimane Dona Ana.

In September of 2013, blood samples were collected from adult ruminants (>12 months) – cattle, sheep and goat in the Zambezia Province in Mozambique (Fig.1). For cattle, 79 samples from two separate farms were tested, and for sheep and goat, the numbers were 145 (five farms) and 141 (six farms) animals, respectively (Fig.1, Table1). Serum was collected from the jugular vein using vacutainer, separated from the whole blood and stored at +4°C until further use.

**Table 1:** Seroprevalence of Schmallenberg virus (SBV) in 1× sera from cattle, sheep and goat collected from eight different farms in Zambezia, Mozambique in September 2013. ‘–’ depicts non-tested farms

	Seroprevalence% (Total no. of samples analysed)							
	Nicoadala Amed	Nicoadala Mingano	Nicoadala Mucelo	Mopeia Chimuara	Mopeia south	Mopeia Deda	Quelimane Dona Ana	Quelimane Padeiro
Cattle	100 (43)	–	–	100 (36)	–	–	–	–
Sheep	43 (7)	–	56 (34)	69 (35)	97 (34)	–	–	49 (35)
Goat	74 (31)	90 (21)	–	89 (28)	–	100 (3)	72 (32)	81 (26)

The serological screening was performed using the ID Screen® Schmallenberg virus Competition Multi-species ELISA (ID-vet, Grabels, France) according to the manufacturer's instructions with undiluted serum. Positive serum samples were diluted 8× to confirm positivity. The choice of an 8× dilution was based on the analytic sensitivity tests performed by ID-vet (internal validation report). Optical reading was done at 450 nm, and the cut-off values suggested by the manufacturer were used, that is, S/N ≤ 40% were considered positive, 40-% < S/N ≤ 50% doubtful and S/N > 50% were considered negative.

## Results and Discussions

All farms investigated had animals that tested positive for SBV antibodies, as summarized in Table1. The ruminant with the highest percentage of SBV-positive animals was cattle. Hundred per cent of the investigated cattle in this study were positive using undiluted sera, and 87% remained positive with the 8× diluted sera. A high SBV seroprevalence in cattle is also seen in different European investigations. In Germany, the within-herd seroprevalence has been shown to be up to 100% (Wernike et al., 2013), and studies from the Netherlands show a within-herd seroprevalence of 70–100% (Elbers et al., 2012). The overall estimated seroprevalence after the initial introduction of SBV has been high in cattle in Europe: 79–94% in France (Zanella et al., 2013), 90.8% in Belgium (Garigliany et al., 2012) and 72.5% in the Netherlands (Elbers et al., 2012). As for cattle, all tested sheep herds had seropositive animals. The within-herd seroprevalence for sheep was, however, lower than in cattle ranging from 43 to 97% between the five investigated farms (undiluted sera). With 8× diluted sera, 71% of the positive samples remained positive, 11% were doubtful and 15% negative. In Belgium, a serological screening for SBV in sheep had a between-herd seroprevalence of 98.03% and a within-herd seroprevalence of 84.31% (Meroc et al., 2013). In the Netherlands, the seroprevalence in sheep has been estimated to 89% (Veldhuis et al., 2013). All investigated farms with goats had goats positive for SBV antibodies, and the within-herd seroprevalence varied from 72 to 100%. Thus, there was in general a higher seroprevalence in goat compared with sheep. This is not in agreement with the results seen from Europe, where sheep herds have displayed a higher seropositivity than goat. A German study presented a within-herd seroprevalence of only 36.7% for goat (Helmer et al., 2013). In the Netherlands, 50.8% of goats were seropositive compared with 89% of sheep (Veldhuis et al., 2013), and in a Belgian study, 40.68% of goats compared with 84.31% of sheep were seropositive (Meroc et al., 2013). This difference in seropositivity between sheep and goat in Belgium and in the Netherlands has been suggested to be a consequence of how the animals were kept with sheep more frequently being kept outdoors (Veldhuis et al., 2013). The farmers, in our study, did not during 2013 observe any clinical signs, such as abortions and malformations, associated with SBV infection or similar virus. This indicates that the exposure may occur continuously as the Zambezia region is characterized by high temperature and humidity enabling vector activity throughout the year.

Although the results from this screening study suggest that SBV might be present in Mozambique, cross-reactivity with another member of the Simbu serogroup cannot be ruled out. Serological cross-reactions between members of the group have been previously described (Kinney and Calisher, 1981). The Simbu serogroup consists of over 25 members, such as Akabane virus (AKAV), Sathuperi virus (SATV), Shamonda virus (SHAV) and Douglas virus (DOUV) (Calisher, 1996). Initial genetic analyses proposed that SBV was a reassortment between SATV and SHAV (Yanase et al., 2012), but further genetic analysis has suggested that SBV may be an ancestor of SHAV. SBV phylogenetically groups with SHAV when analysing the coding regions of the L and S segments, while forming a clade with DOUV and SATV when analysing the M segment (Goller et al., 2012). The ELISA used in this study detects antibodies against the nucleocapsid protein (N) of SBV, encoded from the S RNA segment, and has shown no cross-reactivity with AKAV or Rift Valley fever virus (ID-vet personal communication). Further investigations are needed to identify the actual virus from the host and to determine its genetic relationship with the SBV circulating in Europe.
